# Sexually transmitted infections and semen quality from subfertile men with and without leukocytospermia

**DOI:** 10.1186/s12958-021-00769-2

**Published:** 2021-06-21

**Authors:** Shun Bai, Yuan Li, Yangyang Wan, Tonghang Guo, Qi Jin, Ran Liu, Wenjuan Tang, Meiying Sang, Yuanyuan Tao, Baoguo Xie, Yun Zhao, Wei Li, Xiangdong Xu, Qiuling Yue, Xuechun Hu, Bo Xu

**Affiliations:** 1 Reproductive and Genetic Hospital, Division of Life Sciences and Medicine, The First Affiliated Hospital of USTC, University of Science and Technology of China, 230001 Hefei, Anhui P.R. China; 2 Dermatology Department, The Fifth People’s Hospital of Hainan Province, 570100 Haikou, Hainan, P.R. China; 3 Reproductive Medicine Center, the First Affiliated Hospital of Hainan Medical University, 570102 Haikou, Hainan, P.R. China; 4 Reproductive Medicine Center, The Affiliated Drum Tower Hospital of Nanjing University Medical School, 210008 Nanjing, Jiangsu P.R. China; 5 Department of Urology, Division of Life Sciences and Medicine, The First Affiliated Hospital of USTC, University of Science and Technology of China, 230001 Hefei, Anhui P.R. China

**Keywords:** Leukocytospermia, Sexually transmitted infections, Semen parameters

## Abstract

**Background:**

The role of sexually transmitted infections (STIs) in semen parameters and male infertility is still a controversial area. Previous studies have found bacterial infection in a minority of infertile leukocytospermic males. This study aims to investigate the prevalence of STIs in semen from subfertile men with leukocytospermia (LCS) and without leukocytospermia (non-LCS) and their associations with sperm quality.

**Methods:**

Semen samples were collected from 195 men who asked for a fertility evaluation. Infection with the above 6 pathogens was assessed in each sample. Sperm quality was compared in subfertile men with and without LCS.

**Results:**

The LCS group had significantly decreased semen volume, sperm concentration, progressive motility, total motility and normal morphology. The infection rates of *Ureaplasma urealyticum* (Uuu), *Ureaplasma parvum* (Uup), *Mycoplasma hominis* (MH), *Mycoplasma genitalium* (MG), *Chlamydia trachomatis* (CT), herpes simplex virus-2 (HSV-2) and *Neisseria gonorrhoeae* (NG) were 8.7 %, 21.0 %, 8.2 %, 2.1 %, 3.6 %, 1.0 and 0 %, respectively. The STI detection rates of patients with LCS were higher than those of the non-LCS group (52.3 % vs. 39.3 %), although there was no statistically significant difference between the two groups (*P* = 0.07). All semen parameters were not significantly different between LCS with STIs and without STIs, except the semen volume in the MG-infected patients with LCS was significantly lower than that in the noninfected group.

**Conclusions:**

LCS was associated with a reduction in semen quality, but was not associated with STIs.

## Introduction

Up to 30 % of infertile men have leukocytospermia which refers to the presence of a high concentration (≥ 1 × 10^6^/ml) of white blood cells (WBCs) in semen [1]. Leukocytes, including granulocytes (50–60 %), macrophages (20–30 %) and T lymphocytes (2–5 %), have been shown to be a negative factor for semen quality as they induce low sperm motility [2]. The origin of leukocytes in the ejaculate is mainly from the epididymis and is associated with immunosurveillance [3]. Hence, bacterial and viral infections were considered and detected in semen from symptomatic and asymptomatic leukocytospermic infertile males, although they attending andrology clinics often do not have an apparent infection.

More than 30 sexually transmitted infections (STIs), including herpes simplex virus (HSV), *Chlamydia trachomatis* (CT), *Ureaplasma spp.* (UU), *Mycoplasma hominis* (MH), *Mycoplasma genitalium* (MG) and *Neisseria gonorrhoeae* (NG) have been identified as pathogens that cause genital injury, semen infection, prostatitis, urethritis, epididymitis and orchitis in men [4–7]. Previous studies observed sexually transmitted pathogens in semen in relation to impaired sperm quality and reduced pregnancy rates [8, 9]. However, unlike the strong negative correlation between STIs and fertility in females, the link in males remains controversial [10]. For example, several studies demonstrated that UU increased secondary infertility and reduced sperm quality, including low sperm concentration and motility [11], while other studies did not find a correlation between UU and semen paremeters [12, 13]. Interestingly, UU can be divided into two subtypes, *Ureaplasma urealyticum* (Uuu) and *Ureaplasma parvum* (Uup), and studies have shown that Uup is more associated with poor semen quality than Uuu due to differential pathogenicity [14]. Thus, the controversial results in previous studies may be due to the lack of discrimination between Uuu and Uup.

Recent studies reported that compared with patients with nonleukocytospermia (non-LCS), decreased semen parameters (e.g., sperm concentration, progressive motility, normal morphology) were observed in asymptomatic leukocytospermia (LCS), while those with STI (e.g., UU)-positive leukocytospermia performed more significantly [5, 15]. In this study, we aimed to investigate the prevalence of sexually transmitted pathogens by molecular methods in semen samples from infertile men with and without leukocytes and their effects on semen quality.

## Methods

### Patients

A total of 195 men who sought a fertility evaluation at the Reproductive Center of The First Affiliated Hospital of University of Science and Technology of China (USTC) from July 2019 to July 2020 were included in this study. The exclusion criteria were men who were diagnosed with genetic defects related to reproductive tract, azoospermia, varicocele, testicular trauma, cryptorchidism, postmumps orchitis and chronic severe debilitating medical illnesses. This study was approved by The First Affiliated Hospital of USTC Ethical Committee, Anhui, China (No. 2019P040).

### Semen analysis

The semen volume, sperm concentration, total sperm count, motility, and morphological normality were determined according to the WHO 2010 guidelines for semen analysis (fifth edition, 2010) [1]. Briefly, semen samples were obtained after an abstinence period of 2–7 days. Semen quality was evaluated after liquefaction at 37 °C for at least 30 min. Semen volume was calculated by weighing the semen sample in a tube. Computer-assisted sperm analysis (CASA) was used to measure sperm concentration, progressive motility and total motility (SAS, Beijing, China). Sperm morphology was determined through Diff-Quick staining (Anke Biotechnology, Hefei, China). Leukocytes were stained by peroxidase test using benzidine (Anke Biotechnology, Hefei, China). Antisperm antibodies (AsAs) were investigated by the mixed antiglobulin reaction (MAR) method (Anke Biotechnology, Hefei, China).

Laboratory personnel were trained in semen collecting and detecting procedures according to the National Research Institute for Family Planning. In addition, the laboratory participates in an external quality control (EQA) program by NHC Key Laboratory of Male Reproduction and Genetics.

### Detection of sexually transmitted pathogens

For each male patient, 200 µL of semen specimens was used for the extraction of microorganism DNA. Amplified Sexually transmitted pathogen DNA, including Uuu, Uup (Uup1, Uup3, Uup6, and Uup14), CT, HSV-2, MH, MG and NG, was detected by the “flow-through hybridization” technique using the STD (sexually transmitted disease) 6 GenoArray Diagnostic kit [16]. Samples infected with any pathogen were assigned to the pathogen-infected group, those with no infection with any pathogen were assigned to the non-infected group.

### Statistical analysis

Qualitative variables were described as frequencies (percentages), and quantitative variables were described as the mean ± standard deviation (SD) if normally distributed and medians (interquartile range, IQR) if not. Pearson’s chi-square test and Student’s t-test were used for parametric comparisons, and the Mann-Whitney U test was utilized for nonparametric comparisons. In addition, comparison of sexually transmitted pathogens (Uuu, Uup3, Uup6, MH, MG and CT) were performed by the Kruskal-Wallis test with multiple comparisons. Abnormal semen parameters according to the WHO 2010 recommended standards were listed as follows: semen volume < 1.5 mL, sperm concentration < 15** × **10^6^/mL, progressive motility < 32** × **10^6^/mL and motility morphology < 4 %. *P* value < 0.05 was considered to indicate statistical significance. All statistical analyses were performed using SPSS version 17 (SPSS Inc., Chicago, IL, USA).

## Results

Table 1 displays the mean (± SD) and median (25th, 75th) clinical characteristics and semen parameters from 195 men in the present study. The ages of patients with LCS and without LCS were 32.4 ± 6.9 and 31.6 ± 5.8 years, respectively. Of the studied population, more than 50 % of patients graduated from college/university. A total of 60.0 % of patients were diagnosed with primary infertility. Drinking and smoking were found in 70.3 and 42.6 % of patients, respectively. There was no significant difference between the two groups regarding age, clinical conditions (e.g., BMI, education, duration of infertility, and type of infertility) or lifestyle (e.g., smoking and drinking).

**Table 1:** Characteristics and descriptive statistics of the whole cohort

Clinical characteristics	Total (*n* = 195)	LCS (*n* = 88)	Non-LCS (*n* = 107)	*P*
Age(year), mean ± s.d.	31.9 ± 6.3	32.4 ± 6.9	31.6 ± 5.8	0.39
BMI (kg/m^2^), mean ± s.d.	24.6 ± 3.3	24.1 ± 3.0	24.8 ± 3.5	0.39
Education, n (%)				
primary school	5 (2.6)	1 (1.1)	4 (3.7)	0.32
junior high school	40 (20.5)	21 (23.9)	19 (17.8)	
High school	43 (22.1)	22 (25.0)	21 (19.6)	
College/University	107 (54.8)	44 (50.0)	63 (58.9)	
Duration of infertility, n (%)				
1 year	106 (54.4)	47 (53.4)	59 (55.1)	0.35
2 years	47 (24.1)	25 (28.4)	22 (20.6)	
≥ 3 years	42 (21.5)	16 (18.2)	26 (24.3)	
Type of infertility, n (%)				
Primary	117 (60.0)	49 (55.7)	68 (63.6)	0.26
Secondary	78 (40.0)	39 (44.3)	39 (36.4)	
Alcohol status, n (%)				
Nondrinkers	58 (29.7)	24 (27.3)	34 (31.8)	0.49
drinkers	137 (70.3)	64 (72.7)	73 (68.2)	
Smoking status, n (%)				
Nonsmokers	112 (57.4)	48 (54.5)	64 (59.8)	0.46
Smokers	83 (42.6)	40 (45.5)	43 (40.2)	
Semen parameters				
Abstinence time (days), mean ± s.d	4.3 ± 1.9	4.2 ± 1.9	4.3 ± 2.0	0.75
Semen volume (ml), median (Q1, Q3)	2.9 (2.0, 4.0)	2.8 (1.9, 3.7)	3.0 (2.2,4.1)	0.04
Sperm concentration (**×**10^6^/ml), median (Q1, Q3)	79.0 (37.0, 121.6)	66.4 (28.2, 106.0)	89.5 (54.5, 135.5)	0.01
Progressive motility (%), median (Q1, Q3)	38.0 (18.7, 51.0)	33.8 (19.1, 45.1)	41.6 (16.7, 54.6)	0.02
Total motility (%), median (Q1, Q3)	46.0 (22.4, 59.3)	40.8 (22.5, 55.2)	50.2 (21.4, 63.8)	0.03
Normal morphology (%), median (Q1, Q3)	5.0 (4.0, 8.0)	5.0 (3.0, 7.0)	5.0 (4.0, 8.0)	0.35
leukocytes (×10^6^/ml), median (Q1, Q3)	0.4 (0.1, 4.6)	4.3 (2.9, 6.5)	0.2 (0.1, 0.3)	< 0.001
AsAs (%), median (Q1, Q3)	1.5 (0, 4.0) (n = 124)	1.0 (0, 4.0) (n = 48)	2.0 (1.0, 3.8) (n = 76)	0.99

*BMI* body mass index. Data are presented as frequency (percentage) for categorical variables and mean ± standard deviation (SD) for continuous variables if normally distributed and medians (interquartile range, IQR) if not. *P* values were derived from Pearson’s chi-square test and Student’s t-test for parametric comparisons and the Mann-Whitney U test for nonparametric comparisons. Q1: 25th percentile. Q3: 75th percentile

As expected, the median leukocytes in the LCS and non-LCS groups were 4.3 and 0.2, respectively (Table 2). The median (25th, 75th) semen volume from LCS and non-LCS group were 2.8 (1.9, 3.7) and 3.0 (2.2, 4.1) (*P* = 0.04); sperm concentration were 66.4 (28.2, 106.0) and 89.5 (54.5, 135.5) (*P* = 0.01); progressive motility were 33.8 (19.1, 45.1) and 41.6 (16.7, 54.6) (*P* = 0.02); total motility was 40.8 (22.5, 55.2) and 50.2 (21.4, 63.8) (*P* = 0.03); and normal morphology was 5.0 (3.0, 7.0) and 5.0 (4.0, 8.0) (*P* = 0.35), respectively. The LCS group had significantly decreased semen volume, sperm concentration, progressive motility, total motility and normal morphology. In addition, there were no significant differences between the two groups for AsAs.

**Table 2:** Frequency of STI pathogens in semen samples

Pathogen	Overall (*n* = 195)	LCS (*n* = 88)	Non-LCS (*n* = 107)	*P*
UU	58 (29.2)	25 (28.4)	33 (30.8)	0.71
Uuu, n (%)	17 (8.7)	7 (8.0)	10 (9.3)	0.73
Uup, n (%)	41 (21.0)	18 (20.5)	23 (21.5)	0.86
Uup1, n (%)	4 (2.1)	1 (1.1)	3 (2.8)	0.63*
Uup3, n (%)	19 (9.7)	10 (11.4)	9 (8.4)	0.49
Uup6, n (%)	16 (8.2)	7 (8.0)	9 (8.4)	0.91
Uup14, n (%)	2 (1.0)	0 (0)	2 (1.9)	0.50*
MH, n (%)	16 (8.2)	10 (11.4)	6 (5.6)	0.15
MG, n (%)	4 (2.1)	4 (4.5)	0 (0)	0.04*
CT, n (%)	7 (3.6)	5 (5.7)	2 (1.9)	0.25*
HSV-2, n (%)	2 (1.0)	1 (1.1)	1 (0.9)	0.99*
NG, n (%)	0 (0)	0 (0)	0 (0)	-
Total	87 (44.6)	45 (52.3)	42 (39.3)	0.10

Data are presented as frequency. *P* values were derived from Pearson’s chi-square test if not otherwise indicated

*Fisher’s exact test

As shown in Table 2, the STI-positive semen in subfertility patients was 45.1 %. The STI detection rates of patients with LCS were higher than those of the non-LCS group, (52.3 % vs. 39.3 %), although there was no statistically significant difference between the two groups (*P* = 0.07). Of 195 semen samples, UU accounted for 58 (29.2 %), which was the most prevalent pathogen detected, followed by MH (8.2 %), CT (3.6 %), MG (2.1 %) and HSV-2 (1.0 %). NG was not detected in any semen sample. Strikingly, the rate of MG-positive samples from the LCS group was significantly higher than that in the non-LCS group (*P* = 0.04), while other STI pathogens were similarly detected between the two groups.

Table 3 shows that 13 of the 71 STI-positive samples contained more than one pathogen, including 7 and 6 coinfected samples in the LCS and non-LCS groups, respectively. For the LCS group, 4 and 3 semen samples had 2 and 3 pathogens detections, respectively. Specifically, 5 of 7 semen samples were positive for UU and MH, 3 were positive for UU and MG and 1 was positive for UU and CT. For the non-LCS group, 5 and 1 of these samples were positive for 2 and 3 pathogens, respectively.

**Table 3:** Total simultaneous STI pathogens detected in the LCS and non-LCS groups

Pathogen	Total (*n* = 195)	LCS (*n* = 88)	Non-LCS (*n* = 107)	*P*
No-infect, n (%)	124 (63.6)	52 (59.1)	72 (67.3)	0.24
Infect, n (%)				
1	58 (29.7)	29 (33.0)	29 (27.1)	0.58
2	9 (4.6)	4 (4.5)	5 (4.7)	
3	4 (2.1)	3 (3.4)	1 (0.9)	

Data are presented as frequency. *P* values were derived from Pearson’s chi-square test if not otherwise indicated

The semen parameters from the LCS and non-LCS groups are summarized in Table 4. In both the LCS group and the non-LCS group, semen parameters were similar between patients with STI detection and those without STI detection. Moreover, the prevalence of STI pathogens in patients with normal semen parameters was not significantly different from that in patients with abnormal semen parameters (e.g., semen volume < 1.5 mL, sperm concentration < 15 × 10^6^/mL, progressive motility < 32 × 10^6^/mL, total motility < 40 × 10^6^/mL and motility morphology < 4 %).

**Table 4:** Comparison of semen parameters in patients with LCS and non-LCS

	LCS (*n* = 88)			Non-LCS (*n* = 107)		
	Infected (*n* = 36)	Non-infected (*n* = 52)	*P*	Infected (*n* = 35)	Non-infected (*n* = 72)	*P*
Abstinence time (days), mean ± s.d	4.2 ± 2.0	4.3 ± 1.8	0.81	4.4 ± 2.0	4.2 ± 2.0	0.59
Semen volume (ml), median (Q1, Q3)	2.5 (1.7, 3.4)	2.9 (2.0, 4.0)	0.18	2.9 (2.1, 4.2)	3.1 (2.2, 4.1)	0.92
Semen volume < 1.5 (ml), n (%)	6 (16.7)	4 (7.7)	0.19	2 (5.7)	1 (1.4)	0.25*
Sperm concentration (**×**10^6^/ml), median (Q1, Q3)	53.8 (24.3, 101.4)	69.2 (28.8, 106.8)	0.61	83.8 (38.9, 133.2)	90.2 (55.4, 138.3)	0.55
Sperm concentration < 15 × 10^6^/ml, n (%)	4 (11.1)	6 (11.5)	0.95	2 (5.7)	8 (11.1)	0.49*
Progressive motility (%), median (Q1, Q3)	35.6 (19.9, 45.3)	31.5 (19.0, 45.1)	0.65	46.5 (25.4, 59.2)	40.0 (15.7, 54.4)	0.39
Progressive motility < 32 %, n (%)	14 (38.9)	27 (51.9)	0.23	10 (28.6)	27 (37.5)	0.36
Total motility (%), median (Q1, Q3)	41.2 (23.2, 55.2)	38.2 (22.5, 55.6)	0.72	51.7 (30.2, 63.9)	48.5 (19.4, 63.7)	0.63
Total motility < 40 %, n (%)	16 (44.4)	27 (51.9)	0.49	10 (28.6)	27 (37.5)	0.36
Normal morphology (%), median (Q1, Q3)	5.0 (3.0, 6.8)	5.0 (3.3, 7.0)	0.94	6.0 (4.0, 8.0)	5.0 (4.0, 8.0)	0.29
Normal morphology < 4 %, n (%)	10 (27.8)	13 (25.0)	0.77	8 (22.9)	16 (22.2)	0.94
Leukocytes (×10^6^/ml), median (Q1, Q3)	6.4 (3.0, 6.3)	4.2 (2.6, 7.0)	0.73	0.2 (0.1, 0.3)	0.2 (0.1, 0.3)	0.21

Data are presented as frequency (percentage) for categorical variables and mean ± standard deviation (SD) for continuous variables if normally distributed and medians (interquartile range, IQR) if not. *P* values were derived from Pearson’s chi-square test and Student’s t-test for parametric comparisons and the Mann-Whitney U test for nonparametric comparisons. Q1: 25th percentile. Q3: 75th percentile

*Fisher’s exact test

Table 5 list the association of semen quality and STI pathogens in the LCS group. All semen parameters were not statistically significant for the UU-infected patients, particularly for Uuu-, Uup3- and Uup6-positive patients. Interestingly, multiple comparison analysis found that semen volume was significantly different among groups. Semen volume was lower in patients with MG infection than in those without any STI pathogen detection. Furthermore, leucocytes in semen were not significantly different between the individual STI-positive and STI-negative groups.

**Table 5:** Sexually transmitted infections on semen quality in patients with LCS

	Uuu	Uup3	Uup6	MH	MG	CT	Negative	*P* value^a^
Semen volume (ml), median (Q1, Q3)	2.0 (1.1, 2.9)	3.0 (2.7, 5.1)	2.8 (1.8, 3.6)	2.1 (1.6, 2.8)	1.5 (1.1, 1.8)^b^	2.0 (1.0, 2.5)	2.9 (2.0, 4.0)	0.003
Sperm concentration (× 10^6^/ml), median (Q1, Q3)	35.1 (26.8, 62.0)	60.9 (27.7, 106.1)	110.6 (17.6, 254.6)	45.1 (22.8, 83.2)	48.2 (27.0, 97.7)	46.7 (30.8, 121.5)	69.2 (28.8, 106.8)	0.65
Progressive sperm motility (%), median (Q1, Q3)	33.1 (18.4, 43.2)	30.3 (21.3, 38.6)	32.7 (10.5, 58.0)	34.7 (15.8, 41.6)	40.3 (19.6, 50.1)	39.5 (36.5, 55.4)	31.5 (19.0, 45.1)	0.69
Total sperm motility (%), median (Q1, Q3)	37.7 (19.8, 50.0)	39.6 (27.0, 50.8)	39.1 (12.1, 68.1)	40.2 (19.2, 46.1)	46.0 (22.2, 55.5)	55.2 (44.3, 65.1)	38.2 (22.5, 55.6)	0.56
Normal sperm morphology (%), median (Q1, Q3)	5.0 (3.3, 6.0)	5.5 (2.8, 8.0)	4.0 (2.0, 11.0)	5.0 (4.0, 6.0)	5.5 (3.5, 6.0)	6.0 (3.5, 7.5)	5.0 (3.3, 7.0)	0.99
Leukocytes (×10^6^/ml), median (Q1, Q3)	6.2 (4.1, 11.9)	4.2 (3.1, 6.0)	2.9 (2.4, 8.1)	3.8 (3.1, 6.2)	4.3 (2.4, 6.3)	5.5 (4.0, 9.1)	4.2 (2.6, 7.0)	0.55

Data are presented as medians (interquartile range, IQR). Q1: 25th percentile. Q3: 75th percentile. ^a^*P* value according to the Kruskal-Wallis test. ^b^*P* < 0.05 vs. negative group

## Discussion

During the past decade, the association of STI pathogens, leukocytes and semen quality in male infertility has remained a controversial topic [15]. In this context, we examined the effect of STI pathogenson semen quality in subfertile men with LCS and without LCS using the STD6 GenoArray Diagnostic assay. The method allows the detection of six STI pathogens (NG, CT, UU (Uuu, Uup), MH, MG and HSV-2). The prevalence of STI pathogens in semen samples from men with and without leukocytospermia was 52.3 and 39.3 %, respectively. We also showed that men with MG infection had lower semen volumes than men without infection in the LCS group. However, semen parameters were not significantly different between UU-positive and STI-negative patients.

A wide range of STI-positive percentages were observed in semen samples from infertile men, and this wide range of variability may be due to different types of studied populations, sample sizes and detection methods for STI pathogens [17, 18]. The diagnosis of STIs and therefore therapy is difficult and debated in relation to its association with male fertility [19]. A number of studies have identified the presence of pathogenic bacteria in semen based on culture or PCR [18]. Due to the limitations of culture methods, such as time consumption and low specificity, PCR-based diagnostic methods present rapid and sensitive detection of STI pathogens [20, 21]. In this study, we detected STI pathogens from semen samples by the GenoArray assay, which is a qualitative PCR-based *in vitro* test and rapid identification of mutations related to STD6 in a single test. Hence, this assay could be a potential screening method for semen pathogens in infertility or STD clinics.

HSV infections are associated with abnormal sperm parameters and male infertility by indirectly causing immune responses [22, 23]. Approximately 2–50 % of infertile men were positive for HSV-1/2 DNA [24, 25]. In the current study, the prevalence of HSV-2 pathogens in semen from subfertility patients was 1.0 %, with no difference between the LCS and non-LCS groups. As a low rate of HSV-2 positive semen samples was detected, we did not observe an association of HSV-2 with semen quality.

CT infects the genital tract, resulting in a decrease in seminal volume, sperm concentration, motility and normal morphology [26]. Current studies show that the prevalence of CT is 0.4–42.3 % in asymptomatic males [9]. We observed that the rate of CT-positive semen samples was 3.6 % from subfertility patients. Additionally, CT was associated with a slight decrease in semen volume. The mechanism by which CT affects sperm quality has not yet been elucidated.

NG infection has been associated with urethritis, epididymitis and prostatitis inflammation and thus can lead to infertility. However, the prevalence of NG pathogens was 0-6.5 %, with lower prevalence in infertile men than in men with other STI pathogens [18, 27]. Thus, few studies have investigated its correlation with semen parameters. Similar to previous studies, we detected no NG infection in semen samples from subfertility patients with and without LCS.

UU is the most common microorganism of the lower genitourinary tract in males and is a potentially pathogenic species with an etiologic role in male infertility [28]. The UU-positive rate in the semen samples of infertile men ranges from 5 to 42 %. Historically, the association of CT with semen parameters has been inconsistent. Some of the studies pointed out that UU-positive semen had lower sperm concentrations and sperm motility than UU-negative semen. However, other studies did not observe any correlation between UU and semen parameters [29]. Of interest, our present study confirmed that UU was the most common widespread pathogen in semen (29.2 % of positive samples), with no difference in UU infection between subfertile men with LCS and without LCS. A previous study reported that the frequency of Uuu in the semen of infertile men was higher than that of Uup, indicating that Uup may have a lower correlation with infertility. In this study, we observed a higher prevalence of Uup compared with Uuu in sub-fertile men. Indeed, there was no significant association between semen quality and Uuu and Uup infections. Although we did not find an obvious impact of UU on semen quality, further work exploring the different UU species induced infertility is required.

MH is an STI pathogen that often causes gynecologic and obstetric complications in females, such as postabortion fevers, rupture of fetal membranes, and pelvic inflammatory disease [30]. Although MH is commonly detected in men with nongonoccocal urethritis (NGU), its role in sperm function and male fertility remains unclear. The prevalence of MH in our study was comparable to that reported by Gdoura et al. (8.2 % vs. 9.2 %) [29]. The detection rate of MH was higher in patients with LCS than in those without LCS, although the difference was not statistically significant, suggesting a possible relationship between MH infection and male infertility.

The bacterium MG was isolated and identified as an STI pathogen in two urethral specimens from men with NGU. Studies also showed that the rate of MG infection was higher in symptomatic patients than in asymptomatic patients [31, 32]. Previously, up to 17.1 % of infertile men had MG infection [33], indicating that this pathogen may be important for male fertility outcomes. In line with another study, the prevalence of MG in asymptomatic patients was not high (2.1 %) [34]. In addition, we observed that the MG-positive rate of subfertile men with LCS was higher than that of men without LCS, suggesting a link between the presence of MG pathogens and male infertility. Regarding semen quality parameters, there was a decrease in seminal volume in the sample with MG infection compared to that without MG infection. Therefore, our results confirm that MG infection of the male genital tract could induce infertility. Due to the high azithromycin treatment failure rate (37.4 %) for MG infection in men of infertile couples in China, we should notice that inflammatory changes caused by MG long-term infection result in infertility [33].

According to infertility, men with LCS had a reduction in fructose concentration compared with non-LCS men, and LCS may affect seminal vesicle function. STI pathogens, including MG and CT, are associated with low semen volume, suggesting that subclinical infections seem to affect epididymal function.

There are several strengths of our findings. First, we showed a high rate of STI pathogens in asymptomatic subfertile men. The prevalence of STI pathogens was not associated with the leucocyte counts in semen, thus suggesting that the determination of more than 10^6^ peroxidase- positive cells per milliliter cannot discard STIs and asymptomatic genital tract infections. Second, in this cross-sectional study, we showed that subfertile men with STIs had worse semen volumes than those without infection. Because of the probable impact of infection on semen quality, some tests performed for STI detection in leukocytospermia should be questioned. Thus, screening for STI pathogens could be part of the routine diagnostic workup of subfertile men.

The present study has inherent limitations. First, this study has a possible selection bias because this was a single center-based cross-sectional study. Second, a control group of normal fertile men is lacking. Third, due to a lack of antibiotic therapy against STI pathogens, the potential impact of infection treatment could not be assessed. A final limitation of the study is the small sample size and the results need to be validated with a larger number of patients. Nevertheless, our observations show the importance of investigating semen infection in the clinical practice diagnostic workup of subfertile men.

Overall, we demonstrated that STI pathogens were detected at a high rate in semen samples from asymptomatic subfertile men using a sensitive molecular assay, with no difference in prevalence between the LCS and non-LCS groups. LCS was associated with a reduction in semen quality, but was not associated with STIs. Thus, the present study finding may be beneficial for clinicians to perform an assessment of the association of STIs and subfertile men with LCS.

## Data Availability

The datasets analyzed during this study are available from the corresponding author.

## Abbreviations

STIs: Sexually transmitted infections
LCS: Leukocytospermia
HSV: Herpes simplex virus
CT: Chlamyida trachomatis
UU: Ureaplasma spp.
MH: Mycoplasma hominis
MG: Mycoplasma genitalium
NG: Neisseria gonorrhoeae
Uuu: Ureaplasma urealyticum
Uup: Ureaplasma parvum
NGU: Nongonoccocal urethritis
