# Correction to: Sexually transmitted infections and semen quality from subfertile men with and without leukocytospermia

**DOI:** 10.1186/s12958-022-00940-3

**Published:** 2022-04-19

**Authors:** Shun Bai, Yuan Li, Yangyang Wan, Tonghang Guo, Qi Jin, Ran Liu, Wenjuan Tang, Meiying Sang, Yuanyuan Tao, Baoguo Xie, Yun Zhao, Wei Li, Xiangdong Xu, Qiuling Yue, Xuechun Hu, Bo Xu

**Affiliations:** 1 Reproductive and Genetic Hospital, The First Affiliated Hospital of USTC, Division of Life Sciences and Medicine, University of Science and Technology of China, Anhui 230001 Hefei, P.R. China; 2 Dermatology Department, The Fifth People’s Hospital of Hainan Province, 570100 Haikou, Hainan P.R. China; 3 Reproductive Medicine Center, the First Affiliated Hospital of Hainan Medical University, 570102 Haikou, Hainan P.R. China; 4 Reproductive Medicine Center, The Affiliated Drum Tower Hospital of Nanjing University Medical School, 210008 Nanjing, Jiangsu P.R. China; 5 Division of Life Sciences and Medicine, Department of Urology, The First Affiliated Hospital of USTC, University of Science and Technology of China, Hefei, Anhui 230001 P.R. China

**Correction to: Reprod Biol Endocrinol 19, 92 (2021)**

**https://doi.org/10.1186/s12958-021-00769-2**

Following publication of the original article [1], the authors have reported that affiliations 1 and 5 were incorrectly presented. The correct affiliations are as follows.

Correct:

1. Reproductive and Genetic Hospital, **The First Affiliated Hospital of USTC, Division of Life Sciences and Medicine**, University of Science and Technology of China, Hefei, Anhui, 230001, P.R. China.

5. Department of Urology, **The First Affiliated Hospital of USTC, Division of Life Sciences and Medicine**, University of Science and Technology of China, Hefei, Anhui, 230001, P.R. China.

Incorrect:

1. Reproductive and Genetic Hospital, Division of Life Sciences and Medicine, The First Affiliated Hospital of USTC, University of Science and Technology of China, 230001 Hefei, Anhui, P.R. China.

5. Department of Urology, Division of Life Sciences and Medicine, The First Affiliated Hospital of USTC, University of Science and Technology of China, 230001 Hefei, Anhui, P.R. China.

The original article [1] has been updated.
